# COVID−19, Anti-NMDA Receptor Encephalitis and MicroRNA

**DOI:** 10.3389/fimmu.2022.825103

**Published:** 2022-03-22

**Authors:** Hsiuying Wang

**Affiliations:** Institute of Statistics, National Yang Ming Chiao Tung University, Hsinchu, Taiwan

**Keywords:** anti-NMDA receptor encephalitis, biomarkers, COVID−19, microRNA, vaccine

## Abstract

The coronavirus disease 2019 (COVID-19) pandemic has had an enormous impact on the world, affecting people’s lifestyle, economy, and livelihood. Recently, with the development of vaccines, the number of infected cases has decreased. Many case reports have revealed that COVID-19 may induce other serious comorbidities such as anti-N-methyl-d-aspartate (anti-NMDA) receptor encephalitis. Anti-NMDA receptor encephalitis is an acute autoimmune disease that occurs more commonly in women than in men. To explore the association between COVID-19 and anti-NMDA receptor encephalitis, the microRNA (miRNA) biomarkers of COVID-19, anti-NMDA receptor encephalitis, and other related diseases from the literature are reviewed; then on the basis of these miRNA biomarkers, the relationship between COVID-19 and anti-NMDA receptor encephalitis is discussed. miRNAs are small non-coding RNAs that play important roles in cell differentiation, development, cell-cycle regulation, and apoptosis. miRNAs have been used as biological biomarkers for many diseases. The results in this study reveal that the relationship between anti-NMDA receptor encephalitis and COVID-19 infection or COVID-19 vaccination cannot be excluded; however, the risk that COVID-19 triggers the anti-NMDA receptor encephalitis is not high.

## Introduction

Coronavirus disease 2019 (COVID-19), caused by severe acute respiratory syndrome coronavirus 2 (SARS-CoV-2), has infected more than 173 million people and has had a huge impact all over the world, affecting people’s lifestyle, economy, and livelihood (1). The outbreak of COVID-19 is similar to the prior infectious disease severe acute respiratory syndrome (SARS) that was reported in the early 2000s. SARS was caused by SARS-CoV or SARS-CoV-1 (2). COVID-19 appears to be far more infectious than SARS-CoV (3). Recently, due to the development of vaccines, the number of identified cases has decreased in countries with high vaccination rates. The relationships between daily increasing cases or daily increasing deaths due to COVID-19 and vaccination in 187 countries and regions were studied (4). The results revealed that vaccination can effectively reduce the number of new cases and deaths due to COVID-19. Nevertheless, the vaccines may not be very useful in preventing new variants of SARS-CoV-2; however, they may prevent the risk of developing severe illness.

The origin of SARS-CoV-2 has been widely discussed, and it is more controversial in this sense compared with SARS-CoV-1. It was first identified in Wuhan, China, in December 2019, before rapidly spreading to other countries. Phylogenetic analysis showed that the nucleotide sequences of SARS-CoV-2 samples in China had the lowest diversity, followed by those in Europe and the USA. This result coincided with the transmission order of this virus, whereby SARS-CoV-2 started in China, then broke out in Europe, and finally arrived in the USA (5). SARS-CoV-2 might have been transmitted from bats to humans *via* an intermediary animal such as the pangolin. Bats are the most likely host of SARS-CoV-2 because bat coronaviruses are 96% identical to SARS-CoV-2 at the whole-genome level (6).

COVID-19 has been reported to trigger some other diseases such as autoimmune or autoinflammatory conditions (7). It might trigger a severe inflammatory state known as the “cytokine storm” and the development of thrombotic phenomena, both of which are associated with high mortality. In this paper, the association between COVID-19 and the anti-N-methyl-d-aspartate (anti-NMDA) receptor encephalitis is explored. Anti-NMDA receptor encephalitis is an acute autoimmune disease with a multistage illness progressing from initial psychiatric symptoms to memory disturbances, dyskinesia, seizures, and catatonia (8). It occurs more often in females than in males. The cause of this disease is often unknown. Tumors might induce anti-NMDA receptor encephalitis (9). In addition, virus infection and vaccination might also induce anti-NMDA receptor encephalitis. Vaccination against H1N1 influenza or tetanus, diphtheria, pertussis, and poliomyelitis, or Japanese encephalitis (JE) might induce anti-NMDA receptor encephalitis (10). The association of viruses and various tumors with anti-NMDA receptor encephalitis may account for the comorbidity of these conditions. Anti-NMDA receptor encephalitis may not be diagnosed at an early stage, which may lead to treatment delays and unsatisfactory outcomes.

Several COVID-19 cases associated with anti-NMDA receptor encephalitis have been reported in the literature. A 30-year-old female patient was admitted to the psychiatry department due to paranoid ideation, psychomotor agitation, dysarthria with dysprosody, and visual hallucinations (11). Later, she presented fever, and polymerase chain reaction (PCR) assay results were positive for SARS-CoV-2. In this case, SARS-CoV-2 infection was suspected to trigger the onset of anti-NMDA receptor encephalitis. A 7-year-old boy was diagnosed with anti-NMDA receptor encephalitis associated with COVID-19 without pulmonary involvement or fever (12). A 23-year-old SARS-CoV-2-infected Ecuadorian male patient was hospitalized for psychotic symptoms, and a cerebrospinal fluid (CSF) examination was performed with evidence of anti-NMDA receptor antibodies (13). A 50-year-old man presented an acute onset of psychiatric symptoms, and the test for NMDA receptor antibodies in CSF was positive, while it was negative in serum (14). PCR confirmed SARS-CoV-2 infection. A 23-month-old girl developed fever and movement disorder symptoms, and her PCR test screening for SARS-CoV-2 was positive (15). Her autoantibody testing demonstrated NMDA receptor IgG positivity in the serum and CSF. A 53-year-old woman, during the onset of anti-NMDA receptor encephalitis, had palilalia as a prominent clinical manifestation, accompanied by SARS-CoV-2 infection (16). An 18-year-old girl was admitted to the hospital with generalized tonic-clonic seizures (17). Her CSF PCR was positive for COVID-19, and she also had NMDA receptor antibodies present in the CSF. In addition to these reported cases of COVID-19 infection associated with anti-NMDA receptor encephalitis, a case of anti-NMDA receptor encephalitis triggered by COVID-19 vaccination was reported. A young female presented with acute psychosis, motor dysfunction and a transient bout of aphasia after receiving her first dose of the Pfizer-BioNTech COVID-19 vaccine (18). Later, this patient was diagnosed with anti-NMDA receptor encephalitis by a CSF test. Due to prompt therapies targeting anti-NMDAR antibodies, this patient achieved an excellent clinical outcome.

The virus attaches to a host cell by binding to specific receptor proteins on the cell membrane of the target cell. Different viruses have different binding receptors. SARS-CoV-2 binds the same cell entry receptor, angiotensin-converting enzyme II (ACE2) as SARS-CoV. In addition to these two well-known coronaviruses, SARS-CoV and SARS-CoV-2, another notable coronavirus is the Middle East respiratory syndrome (MERS) coronavirus that binds another cellular receptor, dipeptidyl peptidase 4 (DPP4) (19, 20). The interaction with cell receptors is a key initial step in the life cycle of infectious viruses, and it plays an important regulatory role in viral pathogenesis. Therefore, understanding the molecular interactions of viruses and their receptors can help develop new antiviral therapies and vaccine technologies (21). As a result, it can be concluded that viruses that bind the same receptor may have a more similar pathogenesis than those that bind other receptors. This assumption may apply to other biological mechanisms such as microRNA (miRNA) regulation. In this study, miRNA biomarkers of SARS-CoV-2 and other viruses are reviewed, and the results are used to explore the association between COVID-19 and anti-NMDA receptor encephalitis.

### MicroRNA Biomarkers for COVID-19

miRNAs are small single-stranded non-coding RNAs that play important roles in cell differentiation, development, apoptosis, and modulating gene expression. There are over 2000 miRNAs that have been discovered in humans. miRNAs can be used as genetic biomarkers for various diseases. miRNAs can regulate the protein-coding genes in the human genome (22), and they are well known to be involved in the initiation and progression of many diseases including cancers, neurological diseases, gastroesophageal reflux, and diabetes (23–30). In addition, miRNAs might be important modulators of viral infections and play a role in the treatment of viral infections (31). COVID-19 might use the interplay between miRNAs and other molecules to avoid being recognized and attacked by the host immune protection system, as well as to inactivate related genes that are vital to the immune system (32). SARS-CoV-2 alters self-gene regulation and seizes host miRNAs to provide a protective environment maintaining its latency (33).

miRNA therapy and miRNA biomarkers for COVID-19 infection have been studied (**Table 1**). Honeysuckle is one of the commonly used herbs in traditional Chinese medicine. The absorption of an miRNA in honeysuckle decoction (HD), HD-miR-2911, was demonstrated to inhibit SARS-CoV-2 replication (34). The peripheral blood miRNA levels of 10 patients with COVID-19 and 4 healthy individuals were studied (34). miR‐16‐2‐3p, miR-501‐5p, and miR‐618 were upregulated, while miR‐183‐5p, miR‐627‐5p, and miR‐144‐3p were downregulated in COVID-19 patients. COVID-19 targeted human miRNAs involved in various diseases, with miR-15a, miR-15b, miR-548c, miR-548d, miR-409, miR-30b, and miR-505 exhibiting higher target scores (35). Four miRNAs, miR‐146a‐5p, miR‐21‐5p, miR‐142‐3p, and miR‐15b‐5p, were identified as potential contributors to COVID-19 pathogenesis and might be candidate therapeutic targets (38). The levels of circulating miRNAs of mechanically-ventilated COVID-19 patients and healthy controls were compared (40). Compared with healthy controls, the serum concentration levels of miR-499, miR-21, miR-155, and miR-208a were significantly increased in COVID-19 patients. The circulating expression levels of miR-146a-5p, miR-21-5p, and miR-126-3p were significantly lower in COVID-19 patients compared with gender-matched healthy controls (39). Among the miRNAs in **Table 1**, several were identified by at least two studies. miR‐16‐2, miR-15b, miR-126, miR-146a, and miR-548d were identified by two studies, while miR-21 was identified by three studies (**Table 2**). This reveals that miR‐16‐2, miR-15b, miR-548d, miR-126, miR-146a, and miR-21 could be further investigated as useful miRNA COVID-19 biomarkers. Whereas HD-miR-2911 was also discussed in two studies, it is not a human miRNA.

**Table 1:** COVID-19 studies that investigated miRNAs.

miRNA	Reference
miR‐16‐2, miR‐6501, miR‐618, miR‐183, miR‐627, miR‐144	(34)
miR-15a, miR-15b, miR-548c, miR-548d, miR-409, miR-30b, miR-505	(35)
HD-miR-2911	(34, 36)
miR-4485, miR-483, miR-6891, miR-4284, miR-4463, miR-12136, miR-107, miR-125b, miR-29b, miR-299, miR-501, miR-181, miR-4745, let-7a, miR-374a, miR-194, miR-4454, miR-135b, miR-23b, let-7f-1, miR-429, miR-5701, miR-450b, miR-7-1, miR-26b, miR-23c, miR-374c, miR-374b, miR-26a, miR-365a, miR-365b, miR-940, miR-362, miR-1275, miR-1296, miR-548d, miR‐16‐2, miR-21, miR-155, miR-126	(37)
miR-15b, miR-146a, miR-21, miR-142	(38)
miR-146a, miR-21, miR-126	(39)

**Table 2:** Six COVID-19 miRNA biomarkers identified by at least two studies.

miRNA	Reference
miR‐16‐2	(34, 37)
miR-15b	(35, 38)
miR-548d	(35, 37)
miR-126	(37, 39)
miR-146a	(38, 39)
miR-21	(37–39)

### MicroRNA Biomarkers for Anti-NMDA Receptor Encephalitis and Other Diseases Related to Anti-NMDA Receptor Encephalitis

miRNA biomarkers for anti-NMDA receptor encephalitis and other diseases related to anti-NMDA receptor encephalitis have been discussed in the literature. First, the miRNA biomarkers for anti-NMDA receptor encephalitis were reviewed. So far, only a few studies have investigated miRNA expression for anti-NMDA receptor encephalitis. Zhang et al. recruited anti-NMDA receptor encephalitis patients at the prodromal phase and psychotic phase before immunotherapy, and they studied their blood samples in comparison with a control group (41). The blood samples of five anti-NMDA receptor encephalitis patients and five controls were used for microarray analyses. The miRNAs, let-7a, let-7b, let-7d, and let-7f were found to be significantly downregulated in anti-NMDA receptor encephalitis cases compared to the controls. Next, additional patients and controls were recruited for PCR analysis. The miRNAs let-7a, let-7b, let-7d, and let-7f of the anti-NMDA receptor encephalitis patients were significantly downregulated compared with the controls (see **Table 3**). Finally, they concluded that only let-7b could be used as a biomarker for anti-NMDA receptor encephalitis because the other three miRNAs, let-7a, let-7d, and let-7f, were significantly downregulated in other nervous system diseases compared to the normal controls.

**Table 3:** miRNA biomarkers related to anti-NMDA receptor encephalitis (41).

Disease	miRNA
Anti-NMDA receptor encephalitis	let-7a, let-7b, let-7d, and let-7f

Anti-NMDA receptor encephalitis was found to be related to tumors including ovarian teratoma, testis teratoma, neuroendocrine tumor, small-cell lung cancer, dura mater lesions, and mediastinal teratoma (42–47). The miRNA biomarkers for these tumors were discussed in Wang (2019) (9) (see **Table 4**). In addition, the miRNAs related to viruses and vaccines associated with anti-NMDA receptor encephalitis are listed in **Table 5**. These viruses include the herpes simplex virus (HSV) and JE virus, while the vaccines include those against H1N1 influenza, pertussis, and poliomyelitis.

**Table 4:** miRNA biomarkers for tumors related to anti-NMDA receptor encephalitis (9).

Tumor	miRNA
Ovarian teratoma	miRNA-26b, miRNA-421, miRNA-22, miRNA-492, miRNA-555, miRNA-19a, miRNA-34a, miRNA-620, miRNA-142-3p, let-7a, miRNA-934, miRNA-657, miRNA-720, miRNA-629, miRNA-214
Neuroendocrine tumor	miR-129-5p, let-7, miR-150-5p, miR-29b-3p, miR-22-3p, miR-21-5p, miR-103, miR-107, miRNA-196a
Testis teratoma	let-7a, let-7d, miR-294, miR-371, miR-372, miR-373
Small-cell lung cancer	let-7, miR-27a-5p, miR-34-5p

**Table 5:** miRNAs biomarkers for vaccines or viruses related to anti-NMDA receptor encephalitis (10).

Vaccine or Virus	miRNA
H1N1	miR-323, miR-491, miR-654, miR-10a, let-7c, let-7f, miR-31, miR-29a, miR-148a, miR-146a
Pertussis	miR-202, miR-342, miR-206, miR-487b, miR-576
Poliomyelitis	miR-555
HSV	miR-145, miR-101
JE virus	miR-19b-3p, miR-33a-5p, miR-155, miR-29b, miR-146a

### The Disease Association

**Figure 1:**
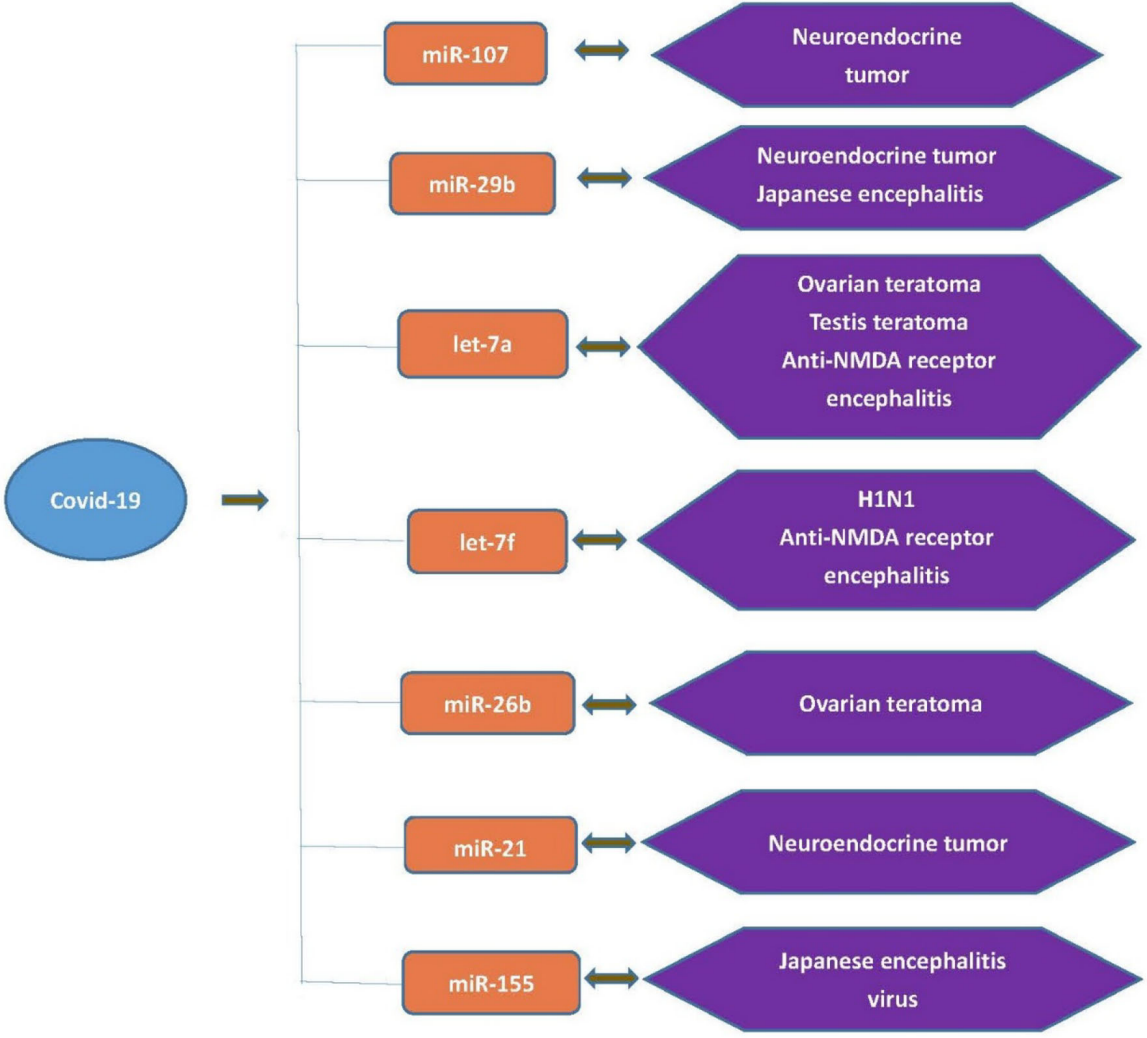
Seven common miRNA biomarkers for COVID-19, anti-NMDA receptor encephalitis, tumors or vaccines related to anti-NMDA receptor encephalitis.

To explore the association between COVID-19 and anti-NMDA receptor encephalitis, the common miRNA biomarkers for COVID-19, anti-NMDA receptor encephalitis, or tumors or vaccines related to anti-NMDA receptor encephalitis were first checked by comparing **Table 1** with **Tables 3**–**5**. Seven common miRNA biomarkers were identified: miR-107, miR-29b, let-7a, let-7f, miR-26b, miR-21 and miR-155 (**Figure 1**).

**Figure 2:**
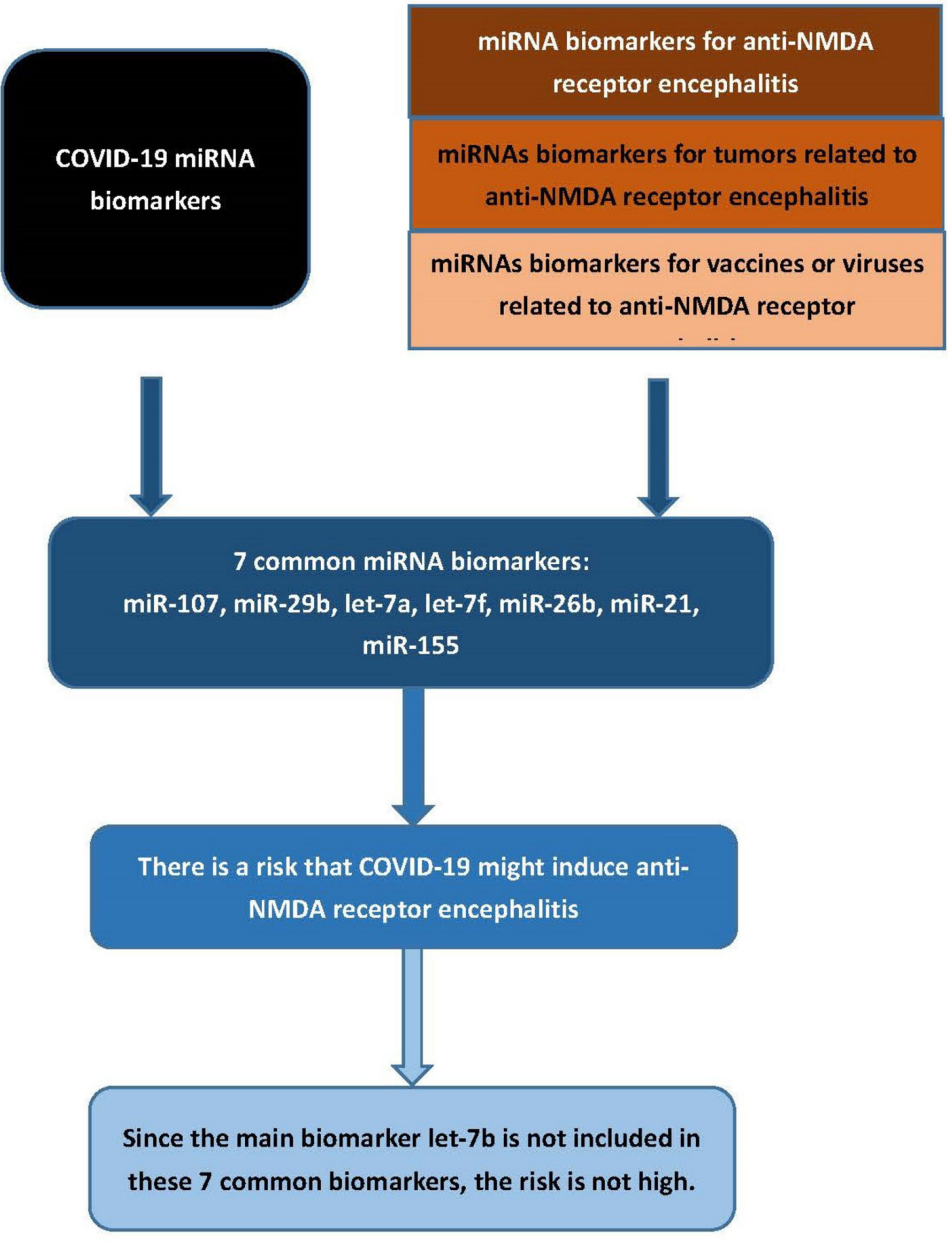
Flowchart of the analysis.

Among these miRNAs, let-7a and let-7f were directly related to anti-NMDA receptor encephalitis (**Table 3**). As mentioned above, although Zhang et al. found that let-7a, let-7b, let-7d, and let-7f were significantly downregulated in patients with anti-NMDA receptor encephalitis compared with the controls, they concluded that only let-7b could be used as the main biomarker for anti-NMDA receptor encephalitis, because let-7a, let-7d, and let-7f were also related to other neurological diseases (41). Several studies reported that miRNA let-7a and let-7f were detected in certain neurological diseases, probably explaining the COVID-19 comorbidity with these conditions. So far, although several cases of anti-NMDA receptor encephalitis triggered by COVID-19 have been reported in the literature, there are not as many as other complications. This may coincide with the fact that the main anti-NMDA receptor encephalitis biomarker, let-7b, was not reported to be a miRNA biomarker of COVID-19. The flowchart of this analysis is provided in **Figure 2**. In addition, two miRNA biomarkers of COVID-19, let-7a and let-7f, were associated with testis teratoma and the H1N1 vaccine, respectively. A 30-year-old male patient with a testicular teratoma and seminoma developed anti-NMDA receptor encephalitis (46). A patient was confirmed to have positive anti-NMDA receptor antibodies after vaccination against H1N1 influenza (48). This may provide indirect evidence showing a relationship between COVID-19 and anti-NMDA receptor encephalitis.

The other five common biomarkers of COVID-19—miR-29b, miR-107, miR-21, miR-155, and miR-26b—were also related to tumors or vaccines associated with anti-NMDA receptor encephalitis. miR-29b was related to both the JE virus and neuroendocrine tumor. miR-107 and miR-21 were related to neuroendocrine tumor. miR-155 was related to JE virus, while miR-26b was associated with ovarian teratoma. This may also provide indirect evidence showing a relationship between COVID-19 and anti-NMDA receptor encephalitis. The relationship between neuroendocrine tumor and anti-NMDA receptor encephalitis has been discussed (44, 49–53). JE vaccination or JE infection has been associated with anti-NMDA receptor encephalitis. A 7-year-old boy was diagnosed with JE, as well as with anti-NMDA receptor encephalitis symptoms (54). Three JE patients who developed relapsing movement or behavioral disorders were examined for anti-NMDA receptor immunoglobulin G (IgG), and positive results were obtained for all the samples (55). Further cases for JE triggering anti-NMDA receptor encephalitis have been discussed (10, 56). Ovarian teratoma has been detected in some female anti-NMDA receptor encephalitis patients (57–61). Patients with ovarian teratomas presented more severe neurological conditions; thus early operative treatment might lead to complete recovery and reduce the risk of relapse.

### Causality

**Figure 3:**
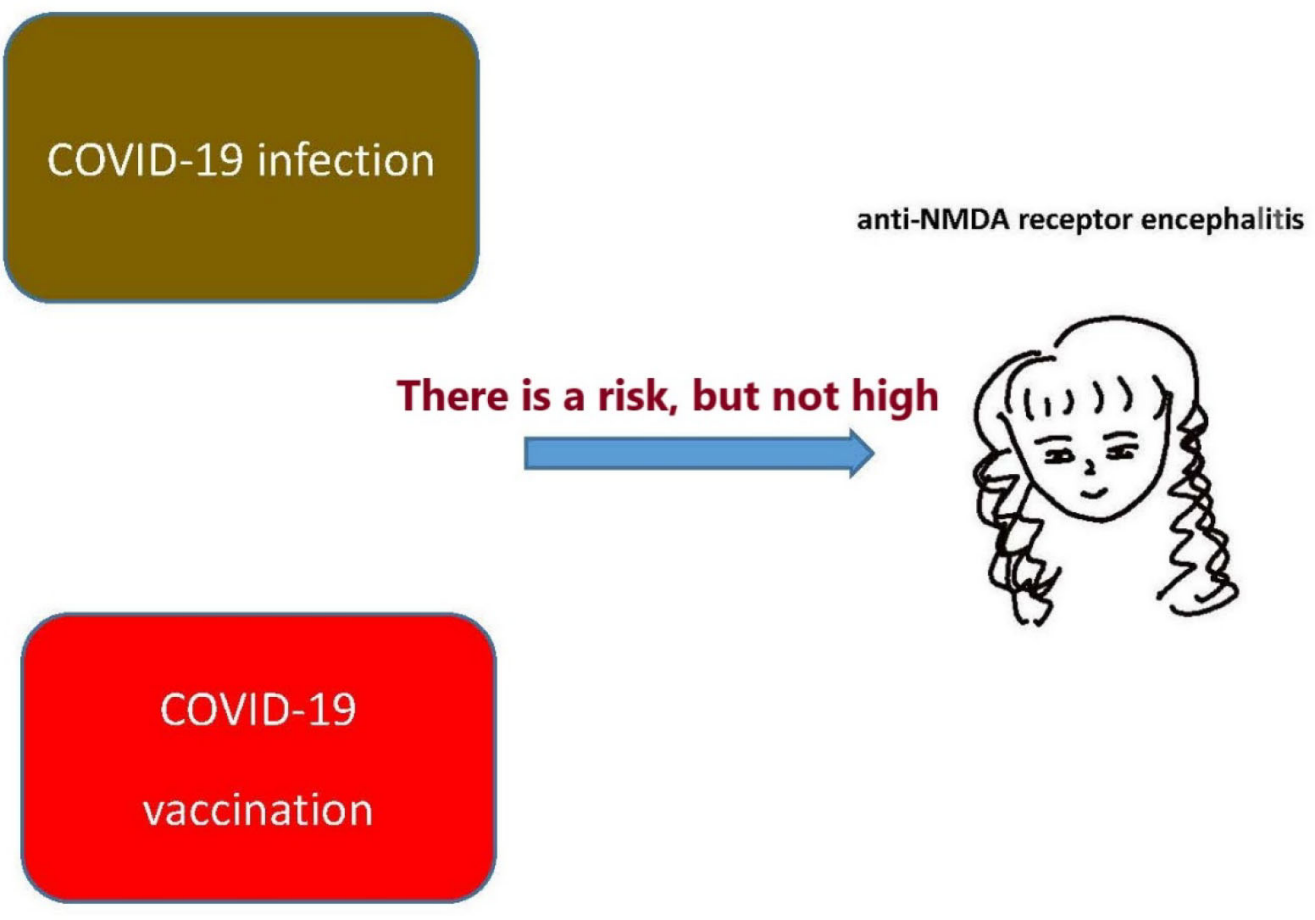
COVID-19 infection or COVID-19 vaccination has a risk of triggering anti-NMDA receptor encephalitis, but the risk is not high.

Only a few cases of COVID-19 triggering anti-NMDA receptor encephalitis have been reported in the literature. This indicates that the risk is not high. **Table 1** presents more than 50 COVID-19 miRNA biomarkers. Among these biomarkers, only seven miRNAs are related to tumors or vaccines associated with anti-NMDA receptor encephalitis, yielding a ratio less than 0.14. This may explain the low risk of developing anti-NMDA receptor encephalitis triggered by COVID-19 infection or COVID-19 vaccination (**Figure 3**).

In spite of the low risk, we may still be interested in investigating the causality of COVID-19 triggering anti-NMDA receptor encephalitis. This can be discussed in several aspects. A molecular mimicry mechanism might contribute to the development of COVID-19-associated neuropsychiatric symptoms (62). Thus, understanding the homology between SARS CoV-2 and human proteins can help explore the mimicry mechanisms during infection. SARS CoV-2 proteins may simulate human proteins, mislead the immune system, and slow down its response. SARS-CoV-2 proteins could interact directly with GABA receptors and NMDA glutamate receptors because of the structural similarities between the NMDA receptor GluN1 and GluN2a subunits with the SARS-CoV-2 nonstructural proteins 8 and 9, respectively (63). As a result, SARS-CoV-2 may induce immune-mediated cross-reactivity to the NMDA receptor.

As, also mentioned above, SARS-CoV-2 can seize host miRNAs to provide a protective environment. It may hijack the miRNA pathway, either by depleting host miRNAs or by producing its own miRNAs. Pawlica et al. discovered a small viral noncoding RNA, named CoV2-miR-O7a, expressed by SARS-CoV-2 (64). CoV2-miR-O7a has the potential to regulate host transcripts by associating with the cellular RNA interference machinery. This finding revealed that SARS-CoV-2 itself could produce miR-like molecules that can influence host gene transcription. These miR-like molecules might resemble miRNAs involved in the function of the NMDA receptor, thereby leading to its dysfunction. In addition, CoV2-miR-O7a was derived from the ORF7a sequence that resembled viral accessory protein ORF7a. Several SARS-CoV-2 proteins including ORF7a, ORF7b, ORF8, and ORF9b are involved in provoking an autoimmune response (65).

These pathological mechanisms might explain the causality of COVID-19 inducing anti-NMDA receptor encephalitis. As these seven miRNAs are common miRNA biomarkers, COVID-19 patients with abnormal expression of at least one of these seven miRNAs might be at a higher risk for anti-NMDA receptor encephalitis.

### Characteristics of COVID-19 Biomarkers

**Figure 4:**
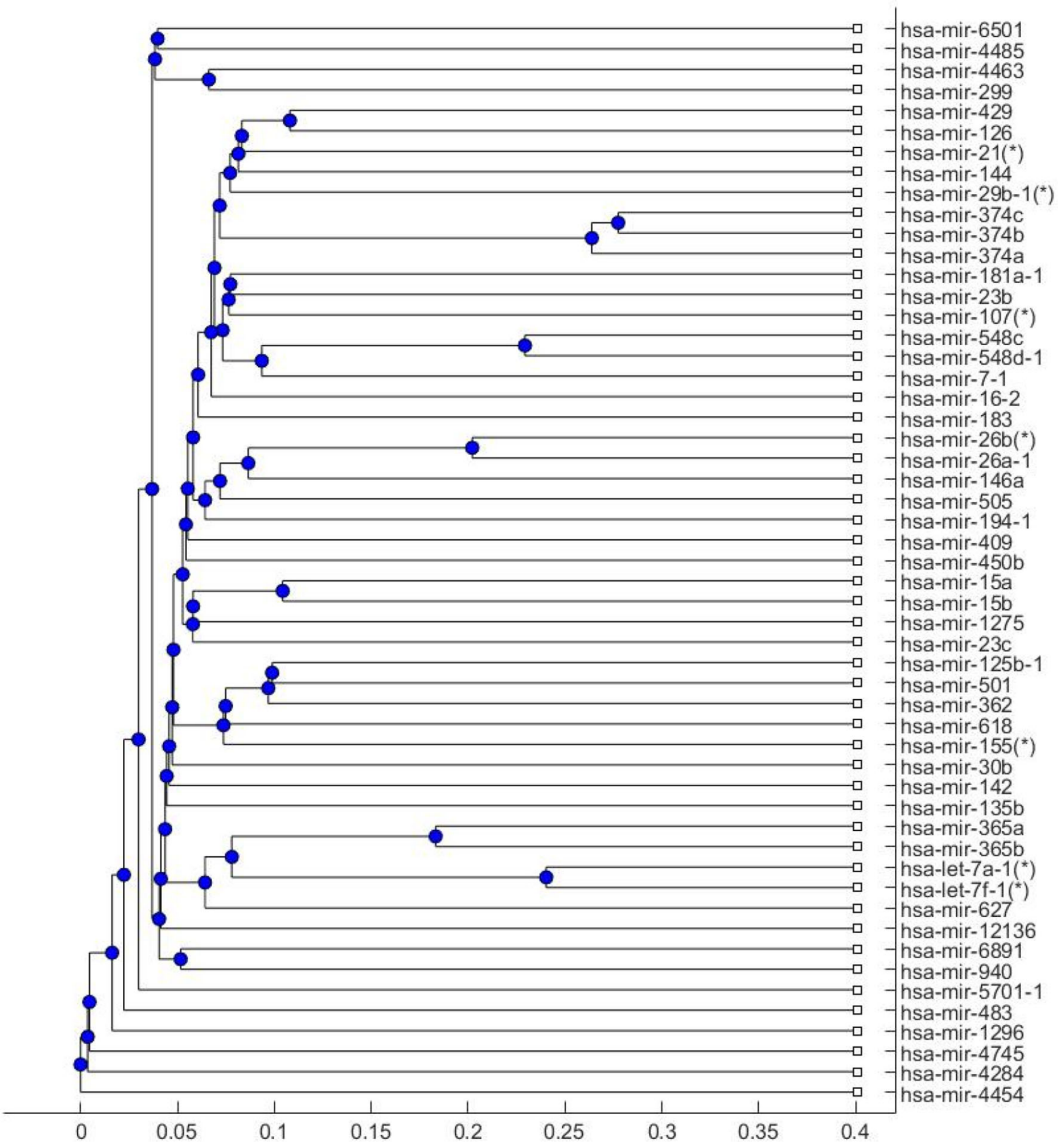
Phylogenetic tree of human COVID-19 miRNA biomarkers. The seven common miRNAs are marked with asterisks.

The seven common miRNA biomarkers in **Figure 1** were used to discuss the risk of COVID-19 infection or COVID-19 vaccination triggering anti-NMDA receptor encephalitis. To examine whether these seven miRNAs can be used as main miRNA biomarkers for COVID-19, a phylogenetic tree for the human COVID-19 miRNA biomarkers in **Table 1** (**Figure 4**) was plotted to study the relationship between these seven biomarkers and other COVID-19 biomarkers. The phylogenetic tree was plotted by Matlab software using the Jukes-Cantor substitution model.

**Figure 4** shows that these seven miRNA biomarkers were all located on the inner branches of the phylogenetic tree. As they are close to most other COVID-19 miRNA biomarkers in **Table 1**, these seven miRNAs can be used to study the association between COVID-19 and anti-NMDA receptor encephalitis.

In addition, most of these miRNA biomarkers of COVID-19 are involved in the inflammatory pathways in COVID-19. Coronaviruses enter the human body through respiratory droplets, fecal-oral transmission, etc. The virus enters the host cell and then releases its RNA genome inside the nucleus before initiating the virus replication. At this stage, the body produces an immune response. However, SARS-CoV-2 infection becomes severe as the inflammatory pathways get activated. The inflammatory pathways involved in COVID-19 include the interleukin-6/Janus kinase/STAT (IL-6/JAK/STAT), the interferon (IFN) cell signaling pathway, the TNFα-NF-κB inflammatory signaling pathway, the JAK/STAT pathway, the toll-like receptor (TLR) pathway, the antibody-mediated pathway, the Bruton tyrosine kinase (BTK) pathway, and the renin-angiotensin system (RAS) pathway (66).

Two of these inflammatory pathways, the JAK/STAT pathway and the RAS pathway, are discussed in this study. The miRTarbase database was used to determine the miRNA biomarkers of COVID-19 involved in these two pathways (67). **Table 6** provides the miRNA biomarkers involved in the JAK/STAT pathway and the RAS pathway. Among the COVID-19 human miRNA biomarkers, only a small number of them were not involved in these two pathways. This reveals that most COVID-19 miRNA biomarkers may play an important role in activating the inflammatory response to SARS-CoV-2 infection. In addition, six of the seven common miRNA biomarkers were involved in both the JAK/STAT and the RAS pathways; the exception was let-7f that was only involved in the JAK/STAT pathway. These inflammatory pathways might contribute to the pathological mechanisms of both COVID-19 and anti-NMDA receptor encephalitis.

**Table 6:** COVID-19 miRNA biomarkers involved in JAK/STAT pathway and Ras signaling pathway.

Inflammatory pathways	COVID-19 miRNA biomarkers involved in the pathway
JAK/STAT pathway	miR‐16‐2, miR‐6501, miR‐618, miR‐183, miR‐144, miR-15a, miR-15b, miR-548c, miR-548d, miR-409, miR-30b, miR-505, miR-483, miR-6891, miR-4284, miR-4463, miR-107, miR-125b, miR-29b, miR-299, miR-501, miR-181, miR-4745, let-7a, miR-374a, miR-194, miR-135b, miR-23b, let-7f-1, miR-429, miR-5701, miR-450b, miR-7-1, miR-26b, miR-374b, miR-26a, miR-365a, miR-365b, miR-940, miR-362, miR-1296, miR-21, miR-155, miR-126, miR-146a, miR-142
RAS signaling pathway	miR‐6501, miR‐183, miR‐627, miR‐144 miR-15a, miR-15b, miR-548c, miR-548d, miR-409, miR-30b, miR-505, miR-4485, miR-483, miR-6891, miR-4284, miR-107, miR-125b, miR-29b, miR-299, miR-501, miR-181, miR-4745, let-7a, miR-374a, miR-194, miR-4454,miR-23b, miR-429, miR-5701, miR-450b, miR-7-1, miR-26b, miR-374c, miR-374b, miR-26a, miR-365a, miR-365b, miR-940, miR-362, miR-1275, miR-1296, miR-21, miR-155, miR-126, miR-146a, miR-142

## Discussion

In this paper, the association between COVID-19 and anti-NMDA receptor encephalitis was discussed. COVID-19 causes many short-term or long-term complications including autoimmune reactions and neurological symptoms. Anti-NMDA receptor encephalitis is not the only complication of COVID-19. As the two COVID-19 miRNA biomarkers let-7a and let-7f are related to other neurological diseases, this may explain why neurological complications other than anti-NMDA receptor encephalitis could be caused by COVID-19.

Many other neurological complications have been reported to be associated with COVID-19 including acute cerebrovascular disease, Guillain-Barré syndrome, and hemophagocytic lymphohistiocytosis (68). A retrospective study investigated 841 patients hospitalized with COVID-19 and found that 57% of patients had neurological symptoms (69). The Spanish Society of Neurology ran a national registry of neurological complications in patients with COVID-19 infection; on the basis of this dataset, a retrospective study investigated patients with encephalopathy or encephalitis (70). A total of 232 patients were recorded in this dataset, including 51 cases of encephalitis or encephalopathy. They concluded that patients with encephalitis were younger than those with other neurological syndromes, but older encephalitis patients had severe encephalopathy and seizures. A child was reported to have encephalitis associated with COVID-19 (71). An 11-year-old boy presented with status epilepticus, and encephalitis was confirmed from the CSF test. His nasopharyngeal swab was shown to be positive for COVID-19. He achieved complete recovery without treatment in 6 days. A Wuhan male had a fever, shortness of breath, and myalgia, with a positive test for SARS-CoV-2 (72). Later, he was diagnosed to have encephalitis. A 79-year-old female was admitted to hospital following a seizure (73). Her second nasopharyngeal sample PCR was positive for COVID-19. The computed tomography (CT) scan and repeat brain MRI suggested limbic encephalitis. A 39-year-old-man presenting with acute encephalitis was diagnosed as having a COVID-19 infection (74). This patient achieved full recovery, suggesting that this condition could be amenable to therapeutic modulation. The effect of plasmapheresis treatment in COVID-19-related autoimmune encephalitis was investigated (75). A 40-year-old woman with type 2 diabetes was admitted to hospital with encephalitis (76). On admission, her nasopharyngeal swab was positive for SARS-CoV-2.

Moreover, many chronic diseases or treatments can increase the risk of COVID-19 infection or increase the risk of developing serious illness from COVID-19. Gastroesophageal reflux (GERD) is a digestive disorder that occurs when acidic stomach fluids back up from the stomach into the esophagus. A GERD drug, proton pump inhibitor (PPI), was shown to increase the risk of COVID-19 infection (77, 78). Collagen could be used as an alternative to treat GERD, as it did not increase the risk of COVID-19 infection (79). Patients with hypertension, cardiac diseases, or diabetes, who receive ACE2-increasing drugs, were found to be at a higher risk of severe COVID-19 infection (80). Obesity was associated with a variety of comorbidities related to the increased morbidity and mortality of COVID-19 (81).

In addition to COVID-19 infection, COVID-19 vaccines have been reported to cause neurological complications. Recently, with the development of vaccines, cases of infection have decreased. Although the vaccines may provide efficient protection against COVID-19, encephalomyelitis, neurological complications, or autoimmune reactions after receiving COVID-19 vaccines have been reported. A 56-year-old female with a history of post-infectious rhombencephalitis developed an acute disseminated encephalomyelitis (ADEM)-like disorder shortly after COVID-19 vaccination (82). Autoimmune hepatitis caused by any vaccination is very rare. Two autoimmune hepatitis cases, a 38-year-old female and a 62-year-old diabetic male, were reported to be triggered by COVID-19 vaccination (83). Two patients with no psychiatric or neurological history presented with encephalopathy and seizures after receiving Moderna COVID-19 vaccines (4). No associations between COVID‐19 vaccination and Bell’s palsy (or Guillain‐Barré syndrome) were found; however, there are special considerations around COVID‐19 vaccines for patients who take immunosuppressive medications (84). On the basis of the results discussed in this paper and the fact that anti-NMDA receptor encephalitis is an autoimmune and neurological disorder, the risk of COVID-19 infection or COVID-19 vaccination triggering anti-NMDA receptor encephalitis cannot be excluded; however, the risk is not high.

## Conclusion

Anti-NMDA receptor encephalitis is an acute disorder. Several cases have been reported to be related to COVID-19 infection. This study investigated miRNA biomarkers to explore the relationship between anti-NMDA receptor encephalitis and COVID-19 infection or COVID-19 vaccination. The results revealed that there were not many common miRNA biomarkers of COVID-19 and anti-NMDA receptor encephalitis-related tumors or vaccines. This may explain why the risk of anti-NMDA receptor encephalitis triggered by COVID-19 infection or COVID-19 vaccines is not high. Nevertheless, there remains a small risk that COVID-19 or COVID-19 vaccination could trigger anti-NMDA receptor encephalitis. Therefore, for COVID-19 patients or people receiving COVID-19 vaccination who develop psychiatric or neurological symptoms, a diagnosis of anti-NMDA receptor encephalitis should be considered if other complications are excluded.

## Data Availability Statement

The original contributions presented in the study are included in the article/supplementary material. Further inquiries can be directed to the corresponding author.

## Author Contributions

HW conceived the presented idea, analyzed the data and wrote the paper.

## Funding

This work was supported by the Ministry of Science and Technology 109-2118-M-009-005-MY2, Taiwan.

## Conflict of Interest

The author declares that the research was conducted in the absence of any commercial or financial relationships that could be construed as a potential conflict of interest.

## Publisher’s Note

All claims expressed in this article are solely those of the authors and do not necessarily represent those of their affiliated organizations, or those of the publisher, the editors and the reviewers. Any product that may be evaluated in this article, or claim that may be made by its manufacturer, is not guaranteed or endorsed by the publisher.
